# Near infrared spectroscopy accurately detects *Trypanosoma cruzi* non-destructively in midguts, rectum and excreta samples of *Triatoma infestans*

**DOI:** 10.1038/s41598-021-03465-8

**Published:** 2021-12-13

**Authors:** Aline Tátila-Ferreira, Gabriela A. Garcia, Lilha M. B. dos Santos, Márcio G. Pavan, Carlos José de C. Moreira, Juliana C. Victoriano, Renato da Silva-Junior, Jacenir R. dos Santos-Mallet, Thaiane Verly, Constança Britto, Maggy T. Sikulu-Lord, Rafael Maciel-de-Freitas

**Affiliations:** 1 Laboratório de Transmissores de Hematozoários, Instituto Oswaldo Cruz, Fundação Oswaldo Cruz, Rio de Janeiro, Brazil; 2 Laboratório de Doenças Parasitárias, Instituto Oswaldo Cruz, Fundação Oswaldo Cruz, Rio de Janeiro, Brazil; 3 Laboratório Interdisciplinar de Vigilância Entomológica de Diptera E Hemiptera, Instituto Oswaldo Cruz, Fundação Oswaldo Cruz, Rio de Janeiro, Brazil; 4 Universidade Iguaçu - UNIG, Rio de Janeiro, Brazil; 5 Laboratório de Biologia Molecular e Doenças Endêmicas, Instituto Oswaldo Cruz, Fundação Oswaldo Cruz, Rio de Janeiro, Brazil; 6 The School of Public Health, The University of Queensland, Herston, QLD 4006 Australia; 7 Instituto Nacional de Ciência e Tecnologia em Entomologia Molecular, Universidade Federal do Rio de Janeiro, Rio de Janeiro, Brazil

**Keywords:** Near-infrared spectroscopy, High-throughput screening, Infectious diseases

## Abstract

Chagas disease is a neglected tropical disease caused by *Trypanosoma cruzi* parasite with an estimated 70 million people at risk. Traditionally, parasite presence in triatomine vectors is detected through optical microscopy which can be low in sensitivity or molecular techniques which can be costly in endemic countries. The aim of this study was to evaluate the ability of a reagent-free technique, the Near Infrared Spectroscopy (NIRS) for rapid and non-invasive detection of *T. cruzi* in *Triatoma infestans* body parts and in wet/dry excreta samples of the insect. NIRS was 100% accurate for predicting the presence of *T. cruzi* infection Dm28c strain (*Tc*I) in either the midgut or the rectum and models developed from either body part could predict infection in the other part. Models developed to predict infection in excreta samples were 100% accurate for predicting infection in both wet and dry samples. However, models developed using dry excreta could not predict infection in wet samples and vice versa. This is the first study to report on the potential application of NIRS for rapid and non-invasive detection of *T. cruzi* infection in *T. infestans* in the laboratory*.* Future work should demonstrate the capacity of NIRS to detect *T. cruzi* in triatomines originating from the field.

## Introduction

American trypanosomiasis, also known as Chagas disease, is a complex anthropozoonosis disease caused by the flagellate protozoan *Trypanosoma cruzi* (Trypanosomatida: Trypanosomatidae), with nearly all domestic and sylvatic mammal species acting as potential reservoirs of the infection. The classical route of parasite transmission is through infected excreta of hematophagous insects belonging to the subfamily Triatominae (Hemiptera: Reduviidae), including 18 genera and 151 species^1,2^. This route of transmission is correlated with the presence of *T. cruzi* infected bugs in domestic and/or peridomestic areas. Globally, Chagas disease affects 21 countries in the Americas, ranging from the southern United States to Argentina^3–5^. In the last decade, Chagas disease has also been reported in Europe, Australia and Asia in travellers and emigrants from endemic areas^6^. The World Health Organization estimates that 6 million people are infected with *T. cruzi*, 70 million people are at risk of getting infected with 12,000 deaths and 30,000 new infections being reported annually^5,7^.

The diversity of triatomine vector species and their habitats are among the factors largely affecting control of the disease in many endemic regions. In particular, three genera, *Triatoma*, *Rhodnius* and *Panstrongylus* are epidemiologically important due to their preferences^8^. In Brazil, there are 66 native species of triatomines^9^. Four of these species; *Triatoma brasiliensis* (Neiva, 1911); *Panstrongylus megistus* (Burmeister, 1835); *Triatoma pseudomaculata* (Corrêa & Espínola, 1964) and *Triatoma sordida* (Stål, 1859) are medically important. These species are also frequently found in sylvatic habitats despite being adapted to domestic and peridomestic areas^10,11^.

Current control measures for Chagas diseases include, among others, the elimination of domestic vector populations through indoor insecticide-spraying^12^ and improving the quality of housing^13^. However, the emergence of insecticide resistance is undermining the current control efforts^14,15^. Additionally, the limited knowledge on the ecology and behaviour of local vectors limits the number of control measures that can be put in place^16^. Entomological surveillance is therefore a critical component of control of Chagas diseases as it determines the appropriate interventions for use. The current need is to develop rapid and effective entomological and epidemiological surveillance systems with capacity to be scaled up to multiple places^14,17^. This surveillance tool will be useful in identifying residual transmission particularly in those countries that have successfully eliminated triatomine vectors or identify reintroduced species in those countries under an elimination phase^18,19^. For countries still under the control phase, surveillance is critical in assessing the effectiveness of interventions that have been put in place.

Surveillance often involves assessing the presence of natural infection in field populations of Triatominae. Traditional techniques for detection of *T. cruzi* in Triatominae include parasite identification by optical microscopy or molecular techniques, such as the polymerase chain reaction (PCR)^20,21^. The detection of *T. cruzi* by microscopy is cost effective, its sensitivity is limited in samples with low parasite load and it is highly dependent on the operator’s expertise; it is a laborious and time-consuming procedure, where fresh insect examination is required for detecting presence of parasite^21–24^. Molecular tools have higher sensitivity and can be performed with dead insects maintained in alcohol solution, but these techniques are costly and require skilled personnel, therefore limiting their application to routine surveillance^21,25^. The development of an alternative tool that is accurate, rapid and cost-effective for the detection of *T. cruzi* infections in Triatominae vectors is an urgent need.

The Near Infrared spectroscopy (NIRS) is a technique that measures the energy absorbed and reflected from a biological sample at specific wavelengths. NIRS is rapid, non-destructive, cost effective, reagent-free and can be used by unskilled personnel following algorithm development^26–29^. Previously, NIRS has been used to non-invasively detect malaria parasites in *Anopheles* mosquitoes^30,31^, the endosymbiont bacteria *Wolbachia* and arboviruses in *Aedes aegypti*^32–35^, discriminate triatomine and *Biomphalaria* species, and age grade mosquitoes^27,29,36–38^.

The aim of the present study was to evaluate the ability of NIRS to non-destructively differentiate *T. cruzi* infected from uninfected triatomines and to detect *T. cruzi* in triatomine’s excreta samples.

## Results

### Prediction of *T. cruzi* in triatomine bugs using spectra collected from the midgut

**Figure 1:**
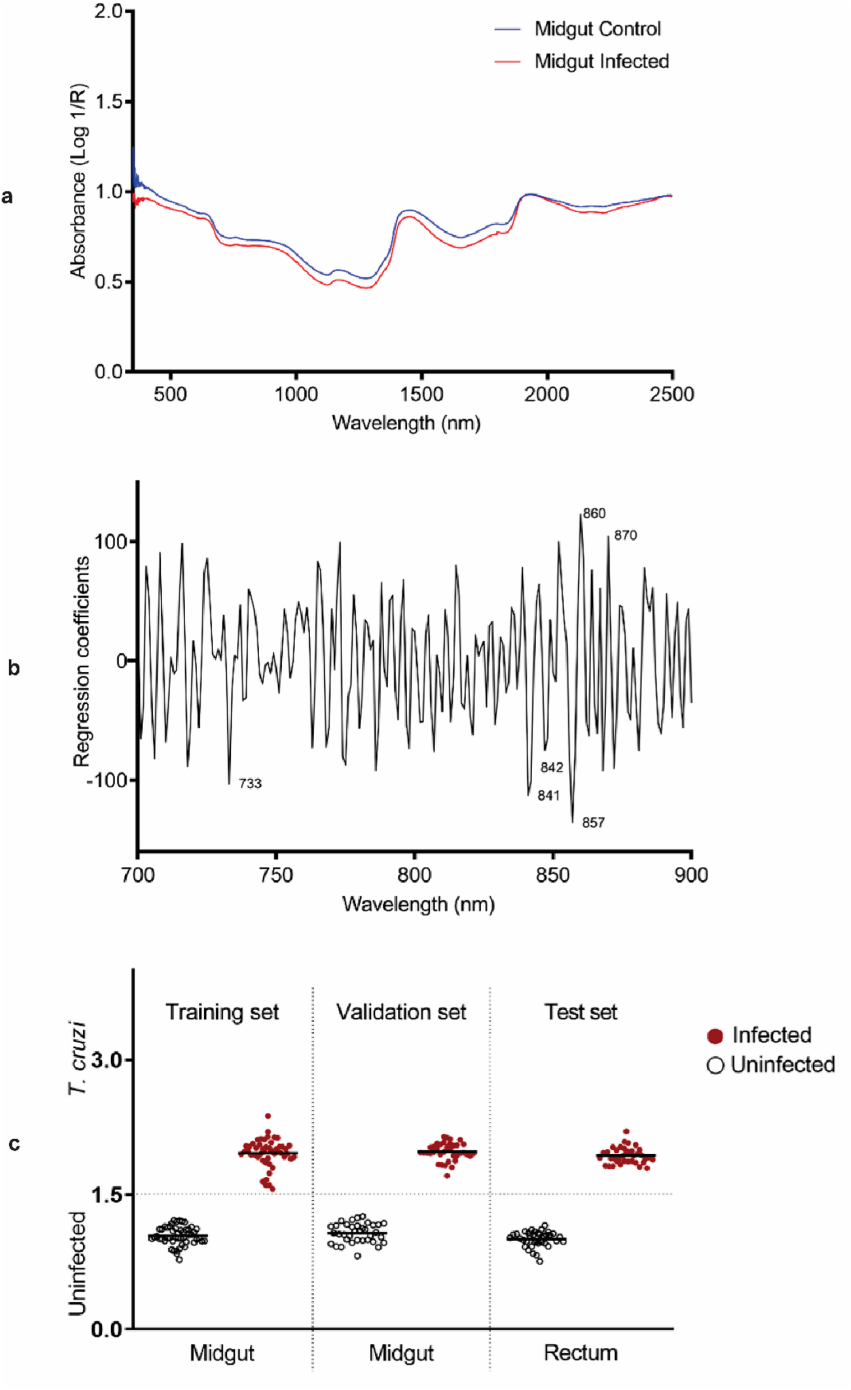
Summary of NIRS data regarding *T. infestans* midgut. (**a**) Average NIR spectra in the 350–2500 nm region from *T. cruzi*-infected and uninfected triatomine midguts. (**b**) Regression coefficients using six factors in the PLS model based on the NIR spectra in the 700–900-nm region for differentiating *T. cruzi*-infected from uninfected samples. (**c**) NIRS differentiation of *T. cruzi*-infected and uninfected triatomines, using leave-one-out cross-validation analysis (training set), prediction of the spectra from the midgut for samples that were excluded from the model (validation set) and prediction of spectra that were collected from the rectum (test set). Each circle represents an individual insect; infection status confirmed by PCR is indicated by red (infected) or empty (uninfected) circles. The lines indicate the mean prediction value for each group. The vertical axis indicates infection status as predicted by NIRS and was drawn by plotting the actual constituent values (1 = uninfected and 2 = infected), with the dotted line indicating the classification cut off point (1.5).

There were noticeable differences between the average raw absorbance spectra of *T. cruzi*-infected and uninfected triatomines that were collected from the midguts. The spectra of uninfected triatomines were observed to have higher absorbance values compared to the spectra of infected triatomines, demonstrating differences in their absorbance characteristics (Fig. 1a). Wavelengths at 733, 841, 842, 857, 860 and 870 nm accounted for the observed differences between infected and uninfected triatomines (Fig. 1b).

Predictive models developed using spectra from midgut differentiated *T. cruzi*-infected from uninfected triatomines with 100% accuracy. When the model was applied to a subset of spectra from the midgut which were excluded from the training model also referred to as the validation set (n = 74), an overall predictive accuracy of 100% was achieved (Fig. 1c). When spectra from the rectum were used to test the model developed using midgut spectra (n = 58), 100% accuracy was achieved (Fig. 1c) (Table 1).

**Table 1:** Sensitivity and specificity of the *T. cruzi* training models.

Experiment dataset	Midgut		Rectum		Dry faeces		Wet faeces	
	%TPR	%SPC	%TPR	%SPC	%TPR	%SPC	%TPR	%SPC
Training dataset	100	100	99	100	98	98	100	100
Validation dataset	100	100	100	100	100	100	100	100

Predicted sensitivity [true positive rate (TPR)] and specificity (SPC) of NIRS are shown for samples used in the training set for midgut (n = 100), rectum (n = 100), dry faeces (n = 100) and wet faeces (n = 100) and samples that were excluded from the training set (validation set) for the same groups (n = 74, 74, 58 and 58, respectively).

### Prediction of *T. cruzi* in triatomine bugs using spectra collected from the rectum

**Figure 2:**
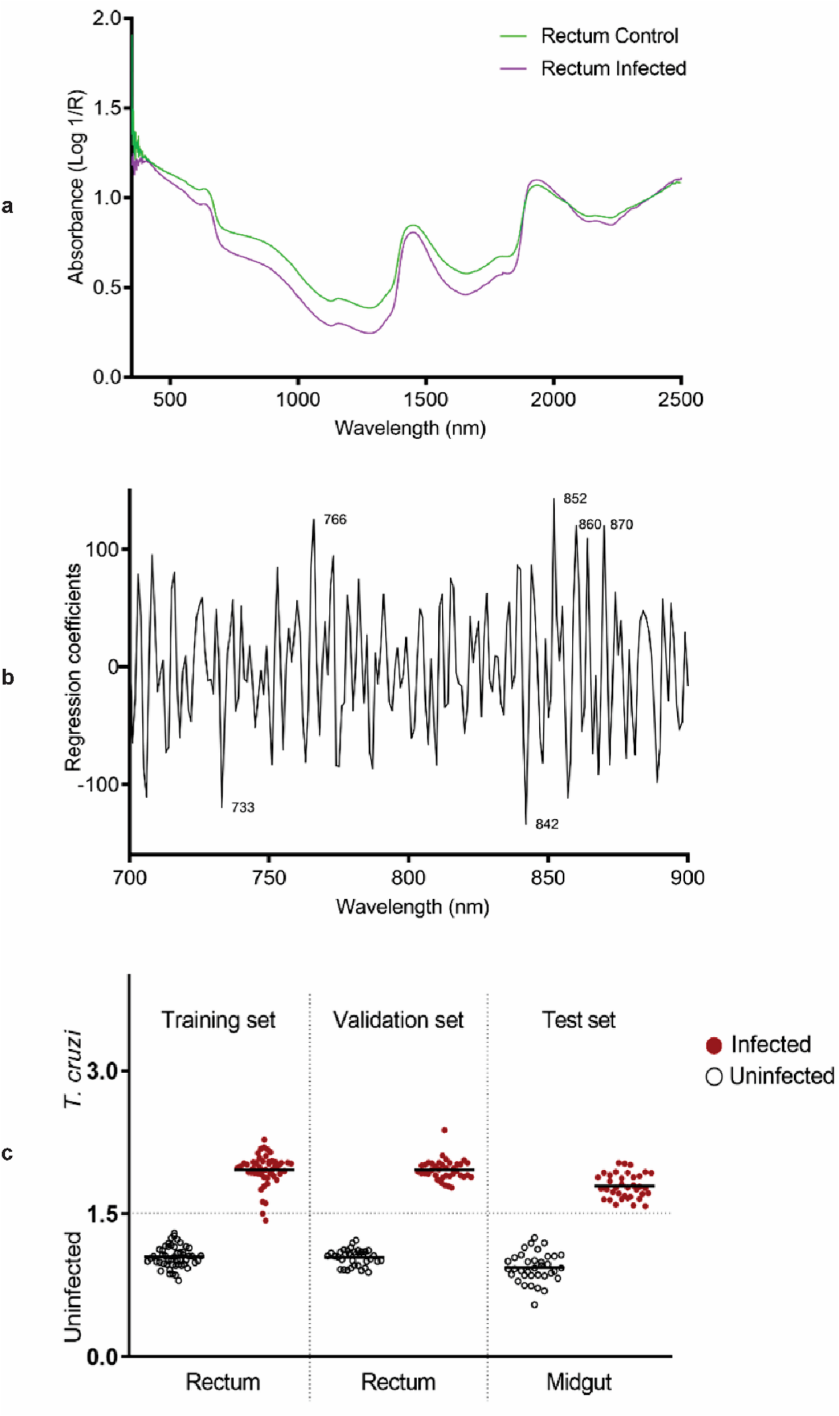
Summary of NIRS data regarding *T. infestans* rectum. (**a**) Average NIR spectra in the 350- to 2500-nm region from *T. cruzi*-infected and uninfected triatomine recta. (**b**) Regression coefficients using six factors in the PLS model based on the NIR spectra in the 700–900 nm region for differentiating *T. cruzi*-infected from uninfected samples. (**c**) NIRS differentiation of *T. cruzi*-infected and uninfected recta, using leave-one-out cross-validation analysis (training set) and samples that were used to validate the model (validation set) and samples and spectra from the midgut that were used to test the model (test set). Each circle represents an individual insect; infection status confirmed by PCR is indicated by red (infected) or empty (uninfected) circles. The lines indicate the mean prediction value for each group. The vertical axis indicates infection status as predicted by NIRS and was drawn by plotting the actual constituent values (1 = uninfected and 2 = infected), with the dotted line indicating the classification cut off point (1.5). Infected triatomines shown below the dotted line and uninfected triatomines shown above the dotted line were falsely predicted.

As observed for the midgut, there were observable differences between the absorbance values of spectral signatures collected from the rectum of the infected and uninfected triatomines. Spectra collected from infected bugs were on average observed to have lower absorbance values compared to spectra of uninfected ones (Fig. 2a). Absorbance peaks 766, 852, 860 and 870 nm contributed to the observed differences between infected and uninfected insects (Fig. 2b). The model developed to predict *T. cruzi* infection in the rectum was 99% accurate for predicting samples that were excluded from the model. Furthermore, this model also predicted infection in midguts with 100% accuracy (Fig. 2c) (Table 1).

### Prediction of *T. cruzi* in triatomine excreta samples

**Figure 3:**
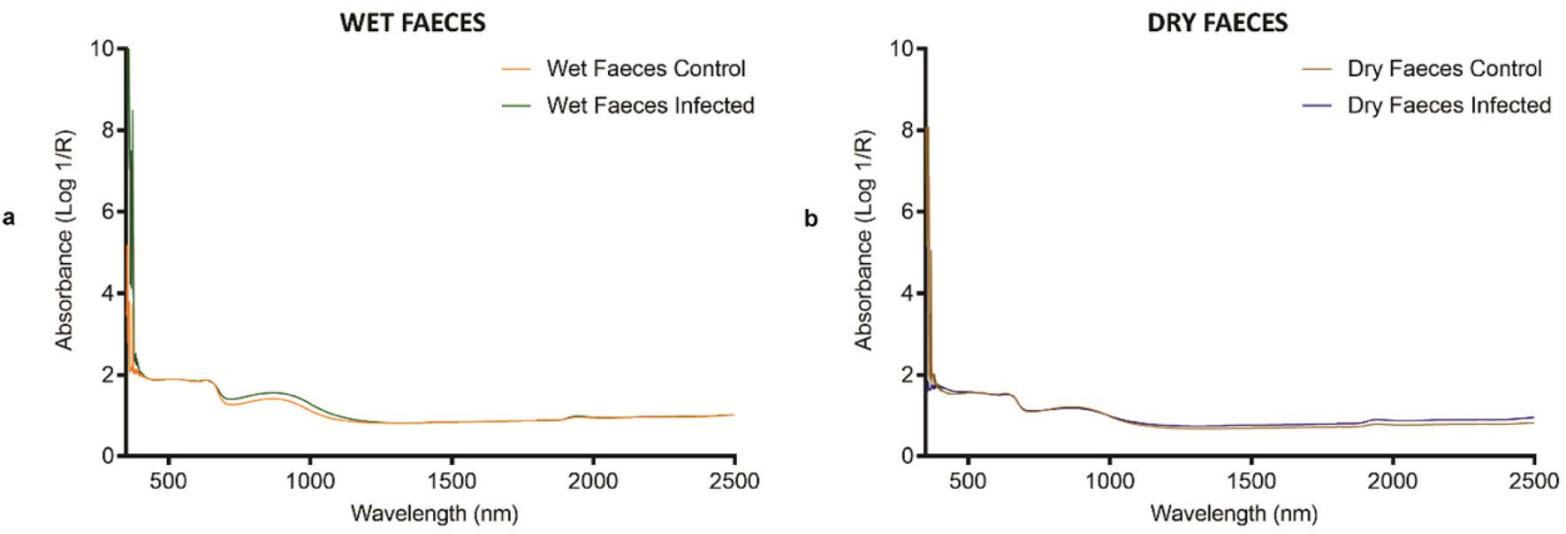
Average NIR spectra in the 350–2500 nm region from *T. infestans* excreta. (**a**) Average NIR spectra in wet faeces (orange for uninfected and green for *T. cruzi-*infected group). (**b**) Average NIR spectra in dry faeces (brown for uninfected and blue for *T. cruzi-*infected group).

**Figure 4:**
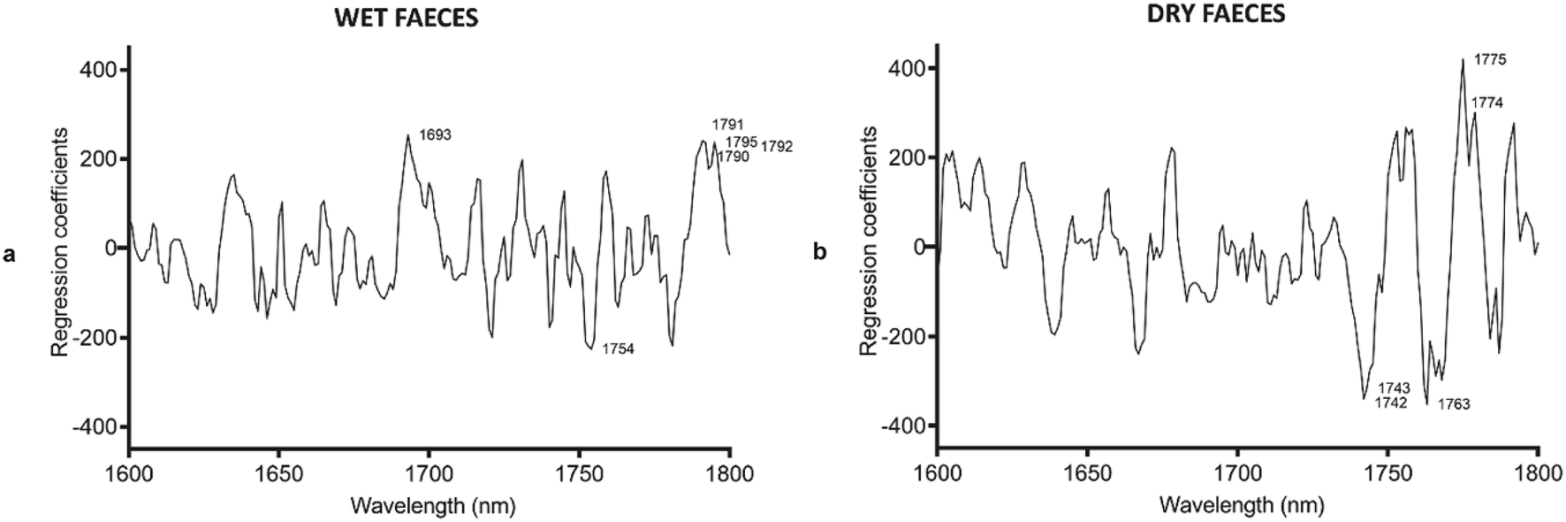
Regression coefficients. Regression coefficients using 6 and 5 factors for (**a**) wet and (**b)** dry excreta, respectively, in the PLS model based on the NIR spectra in the 1600–1800-nm region for differentiating *T. cruzi*-infected from uninfected excreta.

**Figure 5:**
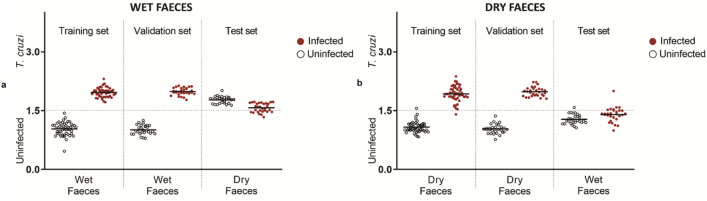
NIRS differentiation of *T. cruzi*-infected and uninfected excreta, using leave-one-out cross-validation analysis. Each circle represents an individual sample; infection status confirmed by PCR is indicated by red (infected) or empty (uninfected) circles. The lines indicate the mean prediction value for each group. The vertical axis indicates infection status as predicted by NIRS and was drawn by plotting the actual constituent values (1 = uninfected and 2 = infected), with the dotted line indicating the classification cut off point (1.5). Infected samples shown below the dotted line and uninfected samples shown above the dotted line were falsely predicted. (**a**) Wet faeces model. (**b**) Dry faeces model.

The average raw spectra of dry and wet excreta and their respective infection status are shown in Fig. 3. Overall, infected excreta samples appeared to have slightly higher absorbance values than uninfected samples. Six and five regression factors were used to differentiate infected excreta samples that were wet and dry, respectively (Fig. 4). The training model developed to predict infection in wet excreta was 100% accurate for samples that were excluded from the model (n = 58). However, the model failed to predict infection in excreta that were dried due to differences in moisture content. Similarly, a training model developed to predict infection in dry excreta was 100% accurate for the validation set (n = 58) but this model could not predict infection status of wet samples (Fig. 5).

## Discussion

Here, we describe for the first time the application of the non-invasively NIRS technique for detecting *T. cruzi* in *T. infestans* in the midguts and in recta as well as in their excreta samples. Our findings represent the first step towards the development of a rapid screening technique for *T. cruzi* to facilitate timely routine surveillance of Chagas disease. Traditional methods used for *T. cruzi* detection in insect vectors are low in sensitivity i.e., optical microscopy, or costly i.e. PCR. Rapid and cost-effective tools will enhance disease surveillance and facilitate timely decisions by policy makers^39^.

*Trypanosoma cruzi* can be identified either in the vector or through its excreta samples^40^. Therefore, the rapid detection of *T. cruzi* by NIRS in two triatomine body parts as well as excreta samples in just a few seconds makes it an ideal tool for surveillance of Chagas disease. Traditional techniques used to identify *T. cruzi* in the vector or excreta samples such as microscopy and PCR require sample processing procedures, technical skills in the case of PCR and can be time consuming. Microscopy can also be very low in sensitivity^22^. Comparatively, NIRS is non-destructive i.e. does not require sample processing procedures, takes 3–5 s to acquire a spectral signature and can be used by unskilled personnel. Although the NIR equipment used in this study (LabSpec 4 I, Malvern Panalytical, Malvern, UK) costs around USD 60,000.00, following the outlay of a spectrometer, no running costs are incurred. One spectrometer can be used for multiple projects over a number of years. NIR has been investigated in several studies and represents the next generation surveillance tool. It has been applied for predicting the age and species of the major African malaria vectors, *Anopheles gambiae* and *An. arabiensis* which are morphologically indistinguishable^27,29^. NIRS has also been used in the lab to predict the age of *Aedes aegypti*^32,41^ and *Ae. albopictus*^38^. More recently, NIRS has been applied to non-invasively detect Zika, Chikungunya and *Wolbachia* inside *Ae. aegypti* mosquitoes^28,34,35,42^ and *Plasmodium* in *An. gambiae*^30^.

The models developed for detecting *T. cruzi* in the midgut predicted the presence of the parasite in the rectum. Similarly, models developed using spectra collected from the rectum were 100% accurate for detecting infection in the midguts. This implies that a single model can be used to predict infection located in either the rectum or the midgut. It also means that spectra can be collected from either the midgut or the rectum. The raw spectra of the midgut and the rectum also appeared similar. The composition of the microbiome of triatomine vectors has been reported to vary throughout the digestive tract. For instance, the relative abundance of gut bacterial community in *T. sordida* varied greatly among the anterior and posterior midgut, and hindgut^43^. The protein profile expression of *T. infestans* digestive tract seems to be affected by the ingestion of blood meal^44^. The varying chemical profile is expected to be reflected in the spectral signature and could overlap with the presence of the pathogen. However, the region analysed was restricted to 700-900 nm which is the third overtone region of NIR region commonly referred to as the optical window. This is the region where most light is absorbed and is the preferrable region for analysing biological samples that have been non-invasively scanned. Despite physiological, biochemical and molecular variations in the triatomine digestive tract during blood feeding and/or *T. cruzi* infection, infected and uninfected triatomines were differentiated with 100% accuracy indicating this part of the NIR region is free of these alterations.

Two additional models were developed to predict infection in the excreta samples. The first model was developed to predict infection in moist/wet excreta, whereas the second model was developed to predict infection in dry samples. Although each model was 100% accurate for predicting the infection status of samples that were excluded from the model, these models were not transferable between wet and dry samples. The presence of water in the wet samples produces water related peaks which are absent in the dry sample. The presence or loss of water/moisture is within the region analysed to predict infection in excreta samples i.e. 1600–1800 nm. Future work should therefore apply separate models depending on the moisture content of the faecal samples. We however highly recommend the use of dried samples as the absence of moisture produces more consistent results. As faecal samples are often found close to places where insects have fed or rested, detection of *T. cruzi* in dried excreta by NIRS is a promising add-on for Chagas disease surveillance in endemic regions. If rapidly detected, the presence of *T. cruzi* in faecal samples located in households could facilitate timely vector control to mitigate parasite transmission or outbreak in an area. Similarly, the microbiome of the insects had no influence on the prediction of infection using excreta samples.

It should be noted that our findings are exclusively based on laboratory experiments under a controlled system. The field application of NIRS to detect *T. cruzi* will most likely require fresh calibration models to account for environmental variations and further investigation on the effect of hindgut microbial composition^45^. Each triatomine species has unique behavioural characteristics and interacts differently with other species and their environment. A universal model incorporating multiple species could therefore be developed for predicting infection in those species. Alternatively, species specific models could be developed for heterogeneous settings.

To the best of our knowledge, this is the first study to report the use of the NIRS technique to predict *T. cruzi* infection in triatomine bugs. Further work to determine NIRS capacity to detect various species of *Trypanosoma* in multiple vectors under a natural setting and its limit of detection is recommended. However, if NIRS is intended to support laboratory-based work, the models developed under this study could be explored further and validated using gold standard techniques preferably molecular-based techniques. Considering the many triatomine species, further work should also focus on assessing the capacity of NIR to detect infections in those species. Such efforts should aim at incorporating NIRS in existing surveillance programs. Additionally, the accuracy of NIRS in other *T. cruzi* lineages or DTUs should be tested to expand its usage and cover the entire genetic variability of the parasite.

## Methods

### Insects rearing

This study was carried out using 200 fifth-instar nymphs of *T. infestans*, a primary vector of *T. cruzi*, obtained from the insectary of the Laboratório de Doenças Parasitárias of Fiocruz, Brazil. Although *T. infestans* is currently restricted to some regions of southern Bolivia, northern Argentina, and residual populations in Brazil, it is historically one of the primary vectors of Chagas disease in Latin America. Insects were maintained under typical insectary conditions: 26 ± 2 °C, 75% humidity. The nymphs were fed weekly on chickens anesthetized with ketamine hydrochloride (10–15 mg/kg) plus xylazine (2 mg/kg) after intramuscular injection (Ethical statement CEUA-IOC L-023/2019). All insects were reared in glass cylinders (23 cm × 11 cm) with paper filters covered with gauze (100 nymphs per glass cylinder).

### *Trypanosoma cruzi* strain

The *T. cruzi* clone used for infection was Dm28c which was obtained from the Oswaldo Cruz Institute Protozoan Collection (code 010), characterized as *Tc*I Discrete Typing Unit (DTU) maintained in NNN (McNeal, Novy & Nicolle) and LIT (Liver Infusion Tryptose) medium. *T. infestans* has previously been found naturally infected with this strains of this DTU^46,47^.

### Experimental infection

Two-hundred *T. infestans* N5 nymphs starved for 25 ± 5 days were used for the artificial blood feeding. Half of them (n = 100), were infected with epimastigotes of *T. cruzi Tc*I (9.4 × 10^7^ parasites/ml) mixed with rabbit blood to form the infected group (Ethical statement CEUA-IOC LW028/2018). The control group (n = 100) was fed on blood without the parasite. Both groups were allowed to feed for 30 min through an artificial feeder^48^. A schematic of the experimental design can be found as Supplementary Fig. S2.

### Scanning of Triatominae

On the 30th day post infection (dpi), the surviving insects (n = 88 for the infected group; n = 86 for the non-infected group) were anesthetized just before scanning by placing them in a closed jar with an acetate-soaked cotton ball for 10–15 s. Then the triatomines were arranged individually with the ventral side up on a spectralon plate (see Supplementary Fig. S1). Insects were scanned with a Labspec 4i spectrometer with 3.2-mm-diameter external probe containing 6 illumination fibres and internal 18.6-W light source (Malvern ASD Panalytical, Boston, MA, USA) as previously described^29^. Two spectra were collected from each triatomine: one from the anterior region of the abdomen (midgut) where epimastigotes are abundant and second spectrum from the posterior region of the abdomen (rectum), where metacyclic trypomastigotes are more abundant.

### Collecting and scanning of excreta

24 h following spectra collection, i.e., 31 dpi, a new blood meal free of parasites was fed to the infected and uninfected insects, thereby inducing excretion. Sampling excreta is of epidemiological relevance because infective forms of *T. cruzi* (metacyclic trypomastigotes) are discarded through insect’s defecation. Following feeding, one faecal drop from each live triatomine bug was obtained by abdominal compression and deposited on a glass slide. Part of the excreta was scanned by NIRS and part of it was preserved at 4–12 °C for PCR analysis. Excreta samples was scanned 3–4 h after the sample was collected and was allowed to completely dry for 4 days before collecting the second spectra.

### Data analyses

The spectra were analyzed in GRAMS Plus/IQ software (Thermo Galactic). The analyses for *T. cruzi* detection in *T. infestans* followed previously published methods^29,34^. Data were subdivided into three groups; (1) training set i.e. samples used for training models, (2) validation set i.e. samples used to test the model and (3) test set i.e. samples that were collected from a different body part or in the case of excreta, samples whose moisture content was different. Four separate models were developed to predict infection in the midgut, rectum, wet excreta and dry excreta. Each training model comprised 100 samples half of which were infected (confirmed through PCR) and the other half were not infected (Table 2). For midguts and recta, spectra were analysed between 700–900 nm whereas spectra of wet and dry faeces were analysed in the region between 1600–1800 nm. Models were developed using Partial Least Square Regression (PLS) using the leave-one-out cross validation where k-fold = 1 i.e. one sample was removed and the remaining samples were used to predict the infection status of the removed sample. The resulting models were applied to a validation and test sets.

**Table 2:** Number of spectra used in training, validation and testing dataset.

	Training dataset				Validation/testing dataset			
	Midgut	Rectum	Wet	Dry	Midgut	Rectum	Wet	Dry
Control	50	50	50	50	36	36	28	28
Infected	50	50	50	50	38	38	30	30
Total	100	100	100	100	74	74	58	58

### Confirmation of *T. cruzi* infection by PCR

24 h following excreta collection, all triatomine bugs were killed and dissected under a stereomicroscope for midguts and recta. Forceps were rinsed in bleach and ethanol between extracting successive samples. Non-infected specimens were used as negative controls during DNA extraction. DNA was extracted from the triatomine gut and excreta using a DNeasy Blood and Tissue Kit (Qiagen). DNAs were stored at − 20 °C until use, and their concentration were measured by Nanodrop 2000c spectrophotometer (Thermo Scientific, Waltham, MA, USA). Conventional multiplex PCR assays were carried out in a final volume of 50 μl, containing: 5 μl DNA (20–25 ng), 5 μl 10 × Taq Platinum buffer, 0.2 mM dNTPs, 4.5 mM MgCl_2_, 1.25 U Taq Platinum DNA polymerase (Life Technologies, Carlsbad, CA, USA), 200 nM 121/122 primers (*T. cruzi* kDNA) and 100 nM P2B/P6R primers (triatomine 12S rRNA gene) as previously described^20^. The triatomine 12S subunit ribosomal RNA gene, acts as an internal control to avoid false-negative results due to the presence of inhibitors elements in the samples, the loss or poor quality of DNA. A positive control for *T*. *cruzi* epimastigotes (strain CL Brener, 10^2^ cells), and a negative control (without DNA) were included in the PCR reaction. PCR samples were loaded onto 2% agarose gels and submitted to electrophoresis at 80 V for 40 min. Gels were stained with a Nancy-520 fluorescent dye (Sigma-Aldrich). The amplification of *T*. cruzi kDNA generates a 330 bp fragment, and a fragment of 163 bp is observed for the triatomine gene.

### Ethical statement

The study was approved by the Ethical Committee for Animal Use of the Oswaldo Cruz Foundation (CEUA-IOC/Fiocruz), through the ethical statements CEUA-IOC L-023/2019 and CEUA-IOC LW028/2018, both described in detail above. Therefore, all experiments were performed in accordance with CEUA-IOC/Fiocruz relevant guideline and regulation. All procedures were conducted in compliance with the ARRIVE (Animal Research: Reporting of In Vivo Experiments) guidelines.

## Supplementary Information

Supplementary Figures.
