# Supplementary material for: Near infrared spectroscopy accurately detects Trypanosoma cruzi non-destructively in midguts, rectum and excreta samples of Triatoma infestans
Source: Sci Rep. 2021 Dec 13;11:23884. doi: 10.1038/s41598-021-03465-8 (PMC8668913; doi:10.1038/s41598-021-03465-8)

SUPPLEMENTARY MATERIAL

Near Infrared spectroscopy accurately detects *Trypanosoma cruzi* non-destructively in midguts, rectum and excreta samples of *Triatoma infestans*

Aline Tátila-Ferreira, Gabriela A. Garcia, Lilha M. B. dos Santos, Marcio G. Pavan, Carlos José de C. Moreira, Juliana C. Victoriano, Renato da Silva-Junior, Jacenir Santos-Mallet, Thaiane Verly, Constança Britto, Maggy T. Sikulu-Lord & Rafael Maciel-de-Freitas

Supplementary Figure S1: Scanning of *Triatoma infestans* nymphs, signaling the areas of spectra collections: the midgut and rectum.

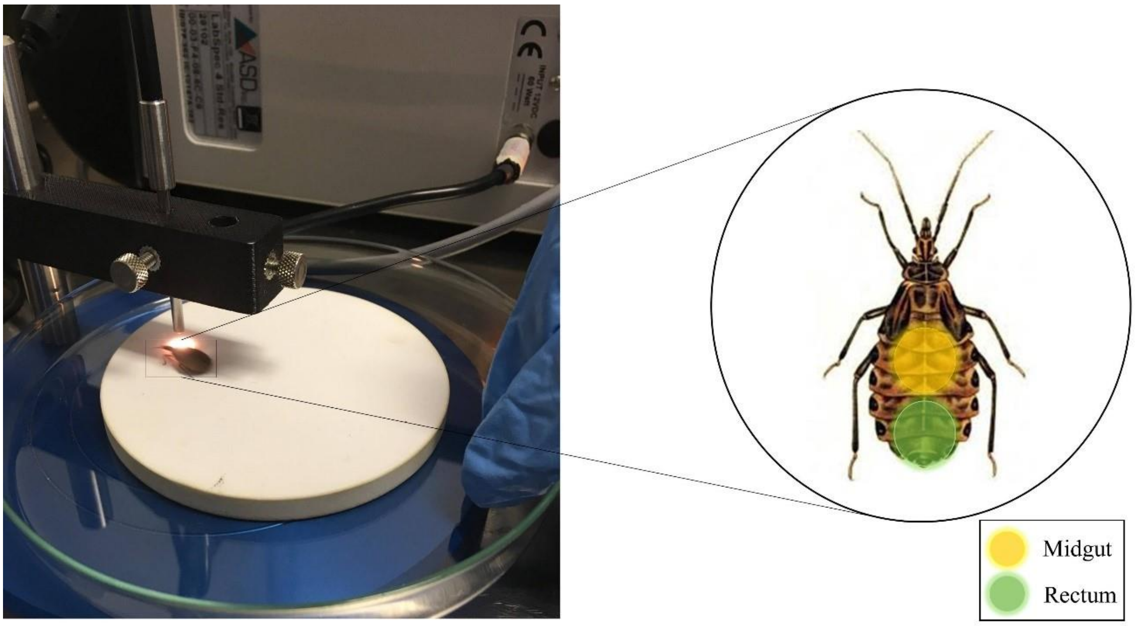

Supplementary Figure S2: Experimental design. DPI: days post infection.

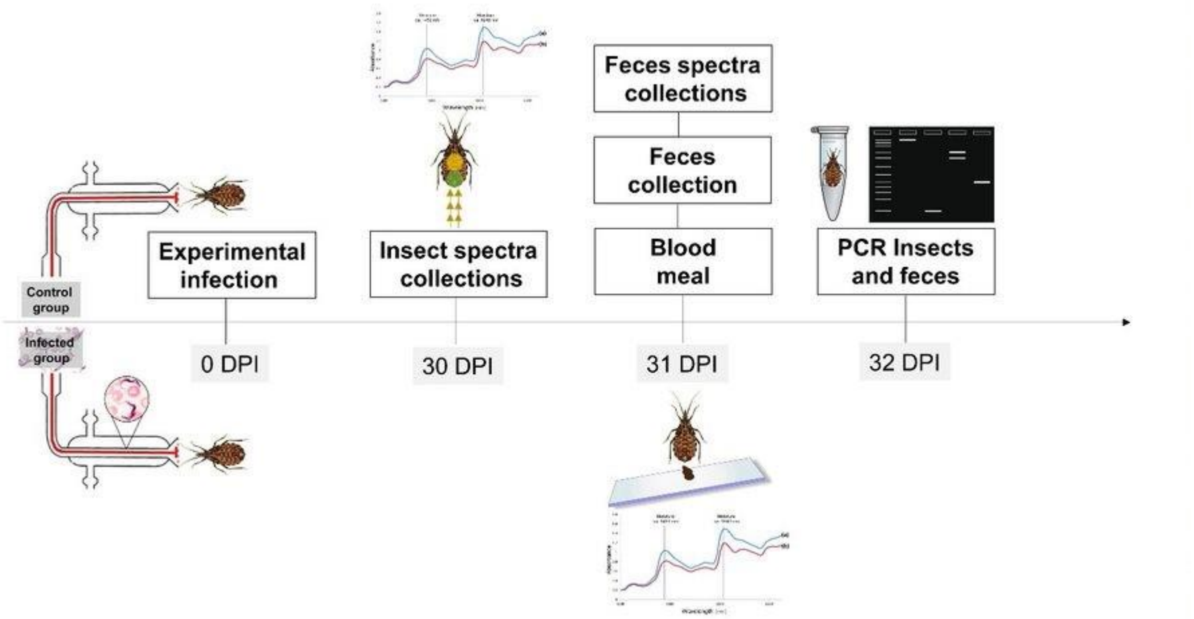

Supplement: Supplementary file 1 — Supplementary Figures. [file 41598_2021_3465_MOESM1_ESM.pdf]
